# Photo-irradiated caffeic acid exhibits antimicrobial activity against *Streptococcus mutans* biofilms via hydroxyl radical formation

**DOI:** 10.1038/s41598-017-07007-z

**Published:** 2017-07-25

**Authors:** Keisuke Nakamura, Midori Shirato, Taro Kanno, Peter Lingström, Ulf Örtengren, Yoshimi Niwano

**Affiliations:** 1 Laboratory for Redox Regulation, Tohoku University Graduate School of Dentistry, 4-1 Seiryo, Aoba-ku, Sendai, 980-8575 Japan; 2 Division of Molecular and Regenerative Prosthodontics, Tohoku University Graduate School of Dentistry, 4-1 Seiryo, Aoba-ku, Sendai, 980-8575 Japan; 3 Department of Cariology, Institute of Odontology, Sahlgrenska Academy, University of Gothenburg, SE-405 30 Gothenburg, Sweden; 4 Department of Clinical Dentistry/Faculty of Health Sciences, The Arctic University of Norway, 9037 Tromsø, Norway

## Abstract

An antimicrobial technique based on photo-oxidation of caffeic acid (CA) has recently been developed, but its effect on biofilm-forming bacteria is unknown. The present study aimed to evaluate the effect of photo-irradiated CA against *Streptococcus mutans* (cariogenic bacteria) biofilm as it relates to hydroxyl radical formation. *S*. *mutans* biofilms grown on hydroxyapatite disks were immersed in CA solution (0–2 mg/mL) and irradiated with LED light at wavelengths of 365, 385, and 400 nm and at irradiances of 500, 1000, and 2000 mW/cm^2^ for 4 min. Biofilm viable bacterial counts were determined by colony counting. The yield of hydroxyl radicals generated by the LED irradiation of CA solution was quantified by electron spin resonance analysis. Of the conditions tested, the highest bactericidal effect, with a > 5-log reduction in viable bacterial counts, was obtained by irradiation of a 1 mg/mL CA solution with 385 nm LED and at an irradiance of 2000 mW/cm^2^. Hydroxyl radical formation was related to this bactericidal effect. The present study suggests that the antimicrobial technique based on the 385 nm LED irradiation of CA is effective against cariogenic biofilms and can be applied as an adjunctive chemotherapy for dental caries.

## Introduction

Improved dental care and increased knowledge of oral diseases as well as associated causative factors of such diseases have brought about a dramatic decrease in the prevalence of caries^1–3^. Accordingly, this improvement has also decreased edentulism leading to an increase in the number of natural teeth in the oral cavities of the elderly^4^. However, since root surfaces are often exposed in this population as a result of gingival recession or periodontal disease, there is a risk for the development of root caries of remaining teeth. Indeed, the prevalence of these lesions is increasing, whereas that of coronal caries has decreased^5, 6^. As society ages, root caries lesions will be a more important issue in the future. Thus, there is still a need to develop a proper control method for these lesions.

Dental caries is an infectious disease caused by acidogenic bacteria in the dental biofilm^7^. *Streptococcus mutans* is the most common acidogenic bacterial species isolated from human cariogenic dental plaques^7, 8^. The bacteria metabolize sucrose not only to produce acid but also to synthesise extracellular polysaccharides (EPS), the main matrix constituent of cariogenic biofilms^9^. This adhesive EPS promotes colonisation by other microorganisms and enhances the biofilm’s resistance to antimicrobials by providing a physical barrier that prevents antimicrobials from entering the biofilm^9–11^. Thus, the primary procedure to deal with these biofilms in dental practice is removal using physical or mechanical techniques such as brushing, flossing, and instrumentation, rather than chemical disinfection. However, proper removal by mechanical means alone is sometimes insufficient particularly at anatomically complex sites. Hence, the application of adjunct antimicrobial chemotherapies could be beneficial in some cases if it is effective against these microbial biofilms^12^.

A novel antimicrobial technique based on photo-oxidation of polyphenols has been developed in our laboratory^13–16^. Exposing an aqueous solution of polyphenols such as caffeic acid (CA), chlorogenic acid, gallic acid, proanthocyanidin, or crude extract of grape to blue light (wavelength: 400 nm) leads to the photo-oxidation of the polyphenolic hydroxyl group. This generates hydrogen peroxide (H_2_O_2_) in the presence of dissolved oxygen^15^. The H_2_O_2_ is subsequently photolysed by the same light, resulting in the generation of hydroxyl radicals^13^, which are more potent oxidants than H_2_O_2_^17^. When the hydroxyl radicals interact with bacterial cells, they cause lethal oxidative damage such as DNA oxidation and lipid peroxidation^13, 18^. Since hydroxyl radicals are generated only at the time and place of light irradiation, the area and antimicrobial activity of the treatment are controllable. In addition, the residual toxicity is likely negligible because the life of the hydroxyl radicals is very short and the remaining polyphenols are edible^13, 19, 20^. Thus, this antimicrobial technique is expected to be applicable to the prophylaxis and/or treatment of dental caries.

It has previously been demonstrated that photo-irradiated CA exhibits higher bactericidal activity against planktonic bacteria including *S*. *mutans* than other polyphenols tested^13^. However, whether this technique can kill bacteria embedded in biofilms remains unknown. It is assumed that biofilms would be more resistant to antimicrobial treatments than planktonic bacteria, as described above. Thus, it will probably be necessary to enhance and optimize the antimicrobial treatment when the target is biofilm-forming bacteria. Application of ultraviolet (UV) light (defined as an electromagnetic waves with a wavelength of <400 nm) may enhance the antimicrobial activity of photo-irradiated CA, as compared to that using visible blue light^16^. Since CA has an absorption peak in the UV region and UV lights has higher photon energy than visible light, the application of UV is expected to enhance both the photo-oxidation of CA and subsequent H_2_O_2_ photolysis^21^, resulting in an increased bactericidal effect. Other methods to enhance bactericidal effects include exposing the biofilm to light at a higher irradiance, using higher concentrations of CA, and extending the treatment time. Based on these considerations, it was hypothesized that the bactericidal activity of photo-irradiated CA would be enhanced by the application of UV (365 or 385 nm) instead of visible blue light (400 nm), and by increasing the irradiance, concentration of CA, and treatment time. Therefore, the present study aimed to evaluate the influence of such treatment conditions on the bactericidal activity of photo-irradiated CA against *S*. *mutans* biofilms in relation to hydroxyl radical formation, in an attempt to optimize the conditions for the application to dental caries treatment.

## Results

### Bactericidal effects of photo-irradiated CA on biofilms

**Figure 1:**
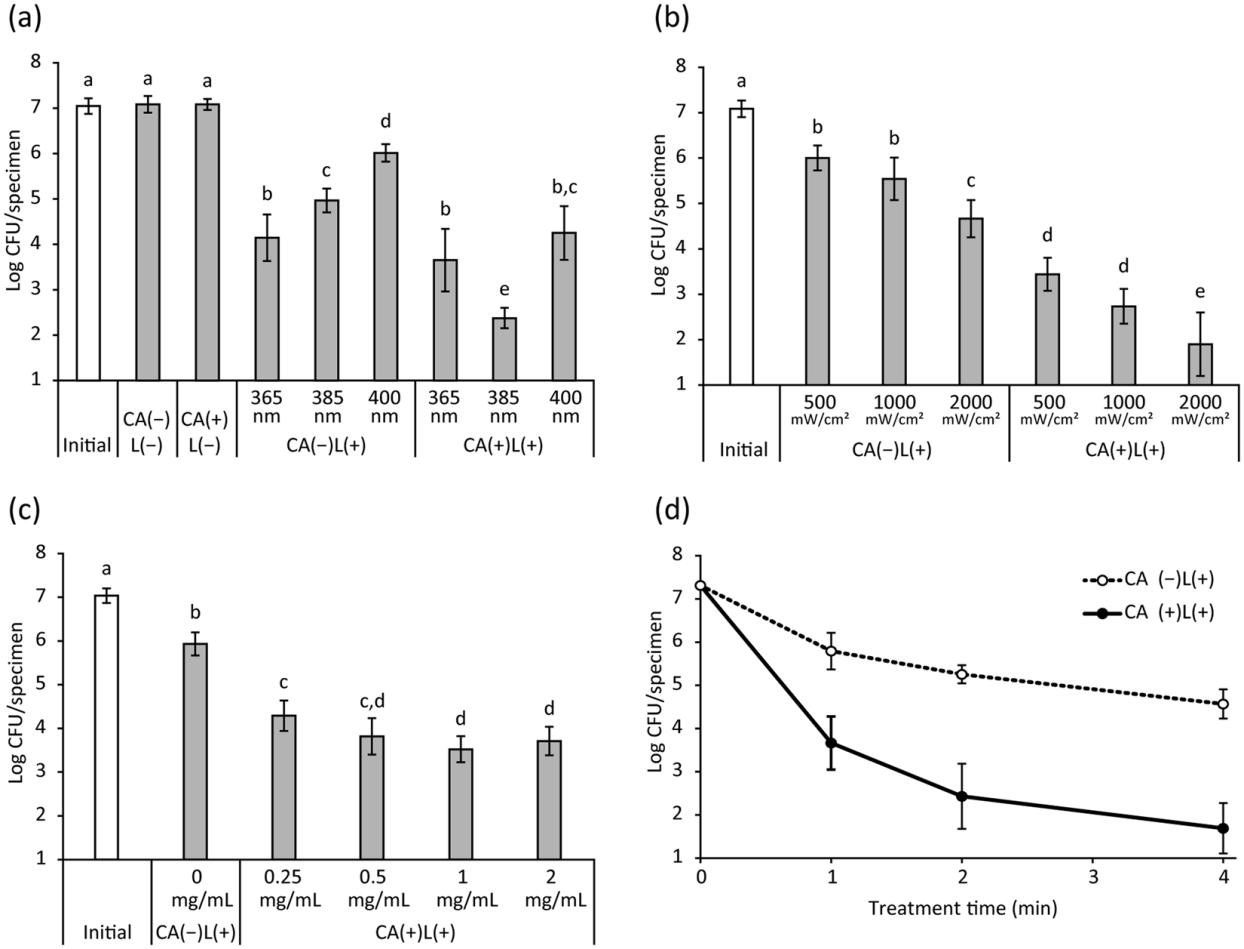
Bactericidal activity of a photo-irradiated caffeic acid (CA) solution against *Streptococcus mutans* biofilms. The bacteria in the biofilm were collected after each treatment by swabbing, and were suspended in saline. The number of bacteria in the suspension was determined by standard plate counting. (**a**) Wavelength-dependent bactericidal activity (irradiance: 1000 mW/cm^2^, concentration of CA: 1 mg/mL, treatment time: 4 min), (**b**) irradiance-dependent bactericidal activity (wavelength: 385 nm, concentration of CA: 1 mg/mL, treatment time: 4 min), (**c**) concentration-dependent bactericidal activity (wavelength: 385 nm, irradiance: 2000 mW/cm^2^, treatment time: 1 min), and (**d**) time-dependent bactericidal activity (wavelength: 385 nm, irradiance: 2000 mW/cm^2^, concentration of CA: 1 mg/mL). Values and error bars indicate the mean and standard deviation, respectively. Different letters above the columns in (**a**), (**b**), and (**c**) refer to significant differences (*p* < 0.05) between different groups. CA(−)L(−): Treatment with pure water in a light-shielding box, CA(+)L(−): Treatment with CA in a light-shielding box, CA(−)L(+): LED irradiation of sample in pure water, CA(+)L(+): LED irradiation of sample in CA.

The results of biofilm bactericidal assays are summarized in Fig. 1. The viable bacterial counts for *S*. *mutans* growing in biofilms on hydroxyapatite were approximately 10^7^ colony forming units (CFUs)/specimen (Fig. 1a). Treatment groups consisted of single or combined treatment using LED irradiation and immersion in CA solution, denoted as CA(−)L(−), CA(+)L(−), CA(−)L(+), or CA(+)L(+). When the biofilm was treated with CA(−)L(−) or CA(+)L(−) (refer to each corresponding figure legend regarding detailed conditions, e.g. wavelength, irradiance, concentration of CA, and treatment time), no significant change in viable counts was observed (Fig. 1a). In contrast, CA(−)L(+) treatment significantly reduced the viable counts depending on the wavelength of light (Fig. 1a). In general, shorter wavelengths resulted in higher bactericidal activity. CA(+)L(+) treatment also significantly decreased the viable counts depending on the wavelength of light, but the influence of wavelength was different from that of CA(−)L(+) (Fig. 1a). In the case of CA(+)L(+), LED irradiation at 385 nm showed the highest bactericidal effect followed by that at 365 nm. Comparing treatments performed at the same wavelength, CA(+)L(+) resulted in significantly lower viable counts than CA(−)L(+), except for when LED irradiation treatment was performed at a wavelength of 365 nm. When comparing immersion in pure water or 1 mg/mL of CA, irradiating the biofilm at 365 nm of LED resulted in similar bactericidal activity. Of the conditions tested, CA(+)L(+) at 385 nm showed the highest bactericidal activity.

The bactericidal effects of CA(−)L(+) and CA(+)L(+) at an LED wavelength of 385 nm were enhanced with an increase in irradiance (Fig. 1b). Comparing treatments performed with the same irradiance, CA(+)L(+) resulted in significantly lower viable counts than CA(−)L(+). Thus, CA(+)L(+) with an irradiance of 2000 mW/cm^2^ was shown to be the optimal condition, based on bactericidal activity.

The effect of the concentration of the CA solution used for CA(+)L(+) treatment on bactericidal activity against the biofilm was limited (Fig. 1c). CA(+)L(+) treatment performed with 0.25 mg/mL CA solution resulted in significantly higher viable counts, compared to counts obtained after treatment with 1 and 2 mg/mL CA solution; however, the difference was less than 1-log CFU. In addition, there was no significant difference in viable counts when comparing treatments performed with 0.5, 1, and 2 mg/mL CA solution.

When the biofilms were treated with CA(+)L(+) at an LED wavelength of 385 nm and an irradiance of 2000 mW/cm^2^, viable counts decreased in a time dependent manner (Fig. 1d). During the first minute of treatment, a 3-log reduction in viable counts was observed. Although the rate of CFU reduction subsequently decreased, a 4-min treatment with CA(+)L(+) resulted in a >5-log reduction of viable counts. In addition, viable counts after a 1-, 2-, or 4-min treatment with CA(+)L(+) were lower than those after CA(−)L(+) treatment.

**Figure 2:**
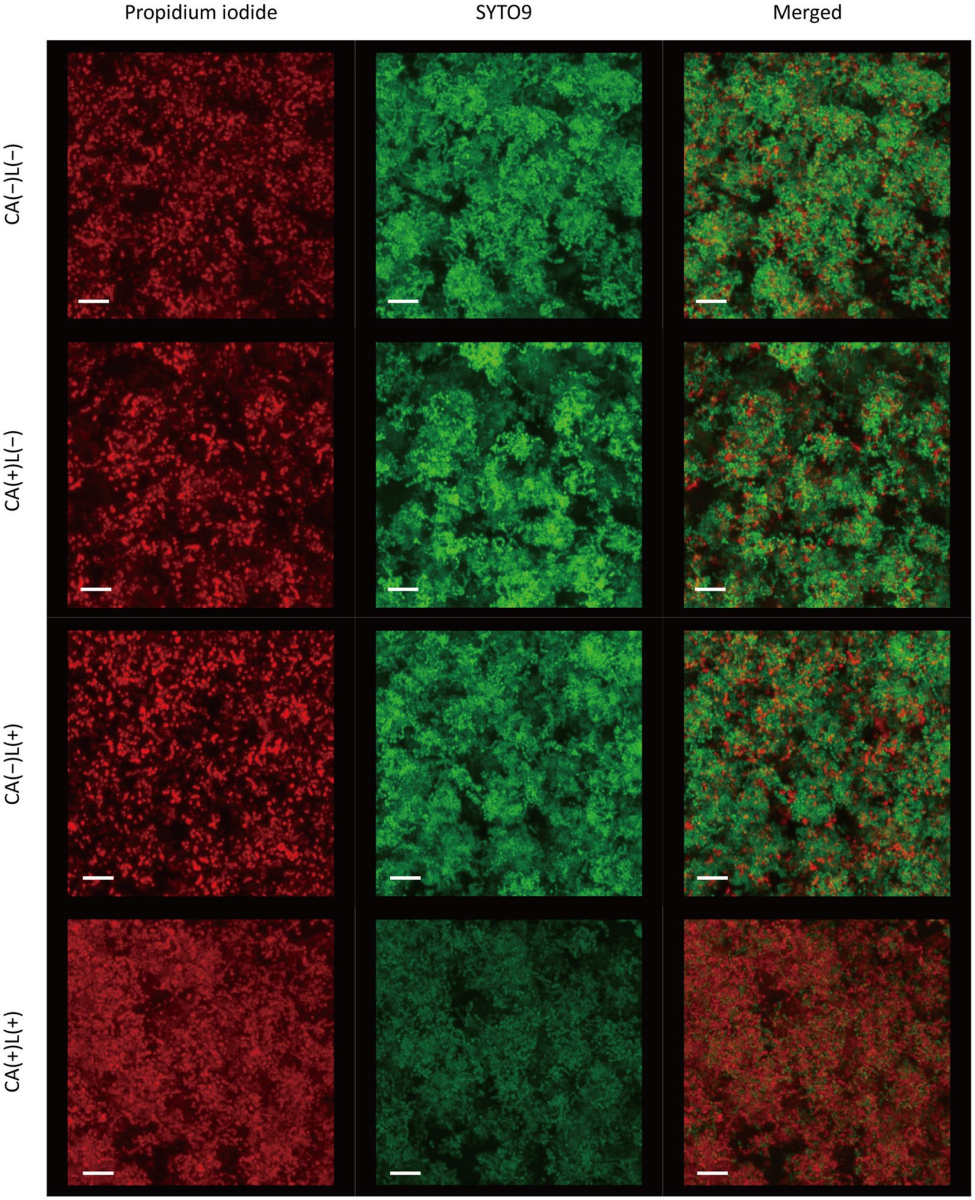
Representative confocal laser scanning microscopic images of *Streptococcus mutans* biofilm treated with photo-irradiated caffeic acid (CA) solution. CA(−)L(−): Treatment with pure water in a light-shielding box, CA(+)L(−): Treatment with CA in a light-shielding box, CA(−)L(+): LED irradiation of sample in pure water, CA(+)L(+): LED irradiation of sample in CA. CA was used at 1 mg/mL and LED irradiation at 385 nm was performed at an irradiance of 2000 mW/cm^2^. Each treatment was performed for 4 min. Dead bacteria were stained by only propidium iodide (red), whereas both dead and living bacteria were stained by SYTO9 (green). Scale bars in each image represent 5 µm.

Representative confocal laser scanning microscopy (CLSM) images are shown in Fig. 2. The biofilm treated with CA(−)L(−), CA(+)L(−), and CA(−)L(+) contained bacterial cells that were stained by SYTO9 and a small number of cells that were stained by propidium iodide (PI). In contrast, the biofilm treated with CA(+)L(+) harboured bacterial cells that were weakly stained by SYTO9 and a large number of cells that were stained by PI. However, the biofilm remained on the specimen even after treatment with CA(+)L(+).

### Bactericidal effect of photo-irradiated CA on planktonic bacteria

**Figure 3:**
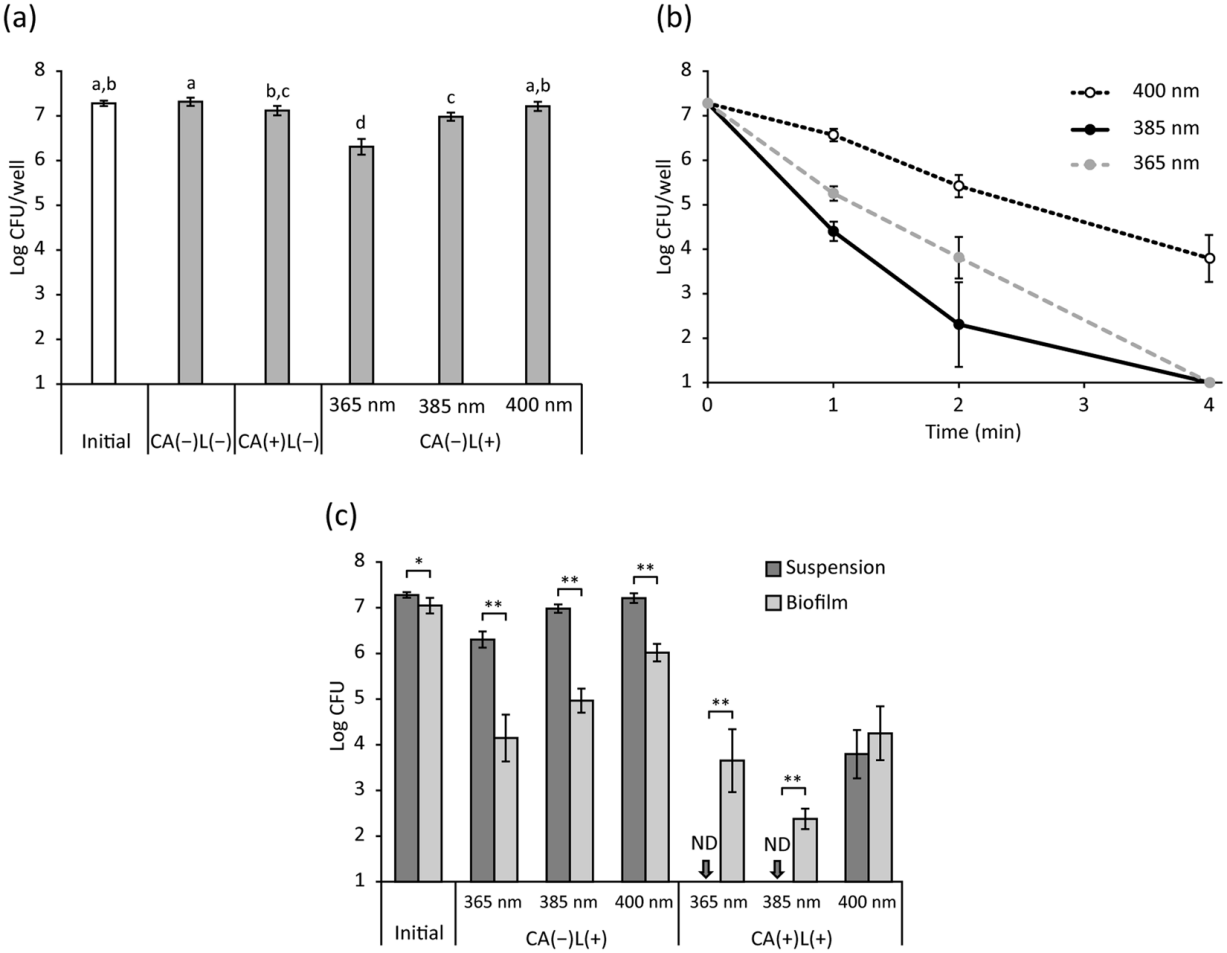
Bactericidal activity of photo-irradiated caffeic acid (CA) solution against planktonic *Streptococcus mutans*. The number of bacteria was determined after each treatment by standard plate counting. (**a**) Bactericidal effect of pure water [CA(−)L(−)], CA [CA(+)L(−)], and photo-irradiation [CA(−)L(+)] (irradiance: 1000 mW/cm^2^, concentration of CA: 1 mg/mL, treatment time: 4 min). (**b**) Time-dependent bactericidal effect of photo-irradiated CA [CA(+)L(+)] (irradiance: 1000 mW/cm^2^, concentration of CA: 1 mg/mL). (**c**) Comparison of bactericidal effects of CA(−)L(+) and CA(+)L(+) against planktonic bacteria and biofilm-forming bacteria (irradiance: 1000 mW/cm^2^, concentration of CA: 1 mg/mL, treatment time: 4 min). Values and error bars indicate the mean and standard deviation, respectively. Different letters above the columns in (**a**) refer to significant differences (*p* < 0.05) between groups. In (**c**), significant differences are shown, *p* < 0.05 (*) and *p* < 0.01 (**). ND: not detected.

Viable bacterial counts of planktonic *S*. *mutans* were not significantly affected by a 4-min treatment with CA(−)L(−) and CA(+)L(−) (Fig. 3a). Although CA(−)L(+) at LED wavelengths of 385 and 365 nm significantly reduced the viable counts from initial levels, the reduction was less than 1-log (Fig. 3a). In contrast, CA(+)L(+) substantially decreased the viable counts in a time dependent manner (Fig. 3b). CA(+)L(+) at 385 nm resulted in the lowest viable counts, and this was followed by treatment at 365 nm, although both treatments decreased viable counts to below the detection limit (>5-log reduction) when performed for 4 min. CA(+)L(+) at 400 nm decreased viable counts by 3.5-log when performed for 4 min. Comparing the resistance of planktonic and biofilm-forming bacteria to treatments performed using the same conditions (irradiance: 1000 mW/cm^2^, concentration of CA: 1 mg/mL, treatment time: 4 min), the former resulted in lower susceptibility to CA(−)L(+), whereas the latter resulted in higher resistance to CA(+)L(+) (Fig. 3c).

### Analysis of hydroxyl radical generation

**Figure 4:**
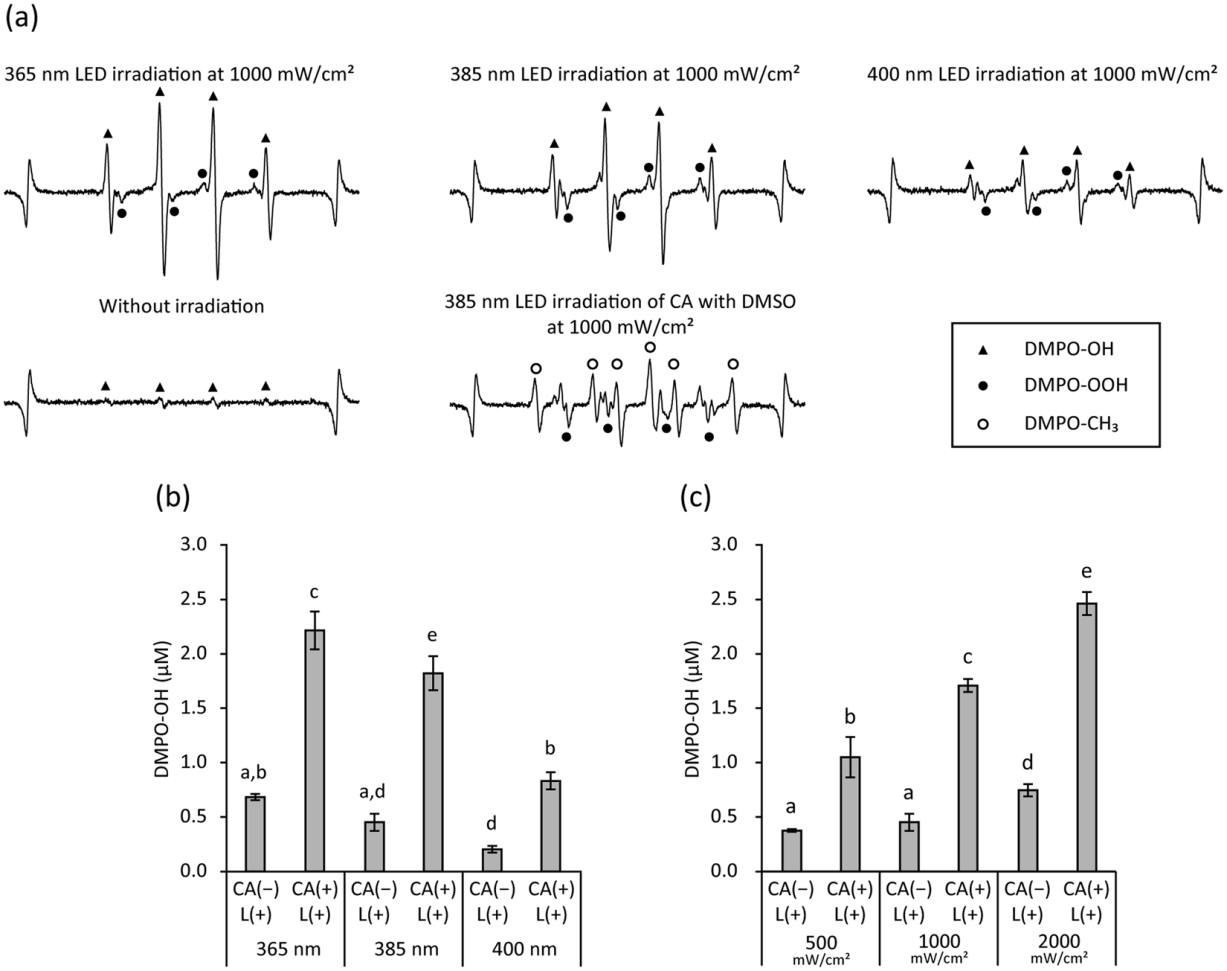
Analysis of hydroxyl radicals generated by LED irradiation of caffeic acid (CA) solution. Hydroxyl radicals were qualitatively and quantitatively analysed using electron spin resonance (ESR)-spin trapping technique using 5,5-dimethyl-1-pyrroline *N*-oxide (DMPO) as a spin trap agent. (**a**) Representative ESR spectra of DMPO-OH (spin adduct of hydroxyl radical: closed triangle) generated using each condition. DMPO-OOH (spin adduct of superoxide anion radical: closed circle) and DMPO-CH_3_ (spin adduct of methyl radical: open circle) were also detected using specific conditions. The peaks at both ends represent Mn^2+^ used as the internal standard. (**b**) Yield of DMPO-OH generated by a 365, 385, or 400 nm LED irradiation of 1 mg/mL CA [CA(+)L(+)] or pure water [CA(−)L(+)] at an irradiance of 1000 mW/cm^2^ for 1 min. (**c**) Yield of DMPO-OH generated by a 385 nm LED irradiation of 1 mg/mL CA or pure water at an irradiance of 500, 1000, or 2000 mW/cm^2^ for 1 min. Values and error bars in (**b**) and (**c**) indicate the mean and standard deviation, respectively. Different letters above the columns in (**b**) and (**c**) refer to significant differences (*p* < 0.05) between different groups.

The representative electron spin resonance (ESR) spectra of 5,5-dimethyl-1-pyrroline *N*-oxide (DMPO)-OH, spin adduct of hydroxyl radical, generated by CA(+)L(+) at different LED wavelengths are shown in Fig. 4a. The presence of DMPO-OH was confirmed by hyperfine coupling constants (hfcc) of a_N_ = a_H_ = 1.49 mT^22^. In addition, a weak signal for DMPO-OOH (spin adduct of superoxide anion radical, hfcc: a_N_ = 1.41 mT, a_H_β = 1.13 mT, and a_H_γ = 0.13 mT) was observed^22^. Without LED irradiation, DMPO-OH occurred at only a trace level (Fig. 4a). When dimethyl sulfoxide (DMSO) was added to the reaction system, DMPO-CH_3_ (spin adduct of methyl radical, hfcc: a_N_ = 1.64 mT, a_H_ = 2.35 mT)^22^ appeared, whereas the signal intensity of DMPO-OH decreased (Fig. 4a). Quantitative analysis revealed that the yield of DMPO-OH generated by CA(−)L(+) and CA(+)L(+) depended on the wavelength and the irradiance (Fig. 4b,c). In general, shorter wavelengths and higher irradiances resulted in larger yields. In addition, CA(+)L(+) generated significantly larger yields of hydroxyl radicals than CA(−)L(+).

**Figure 5:**
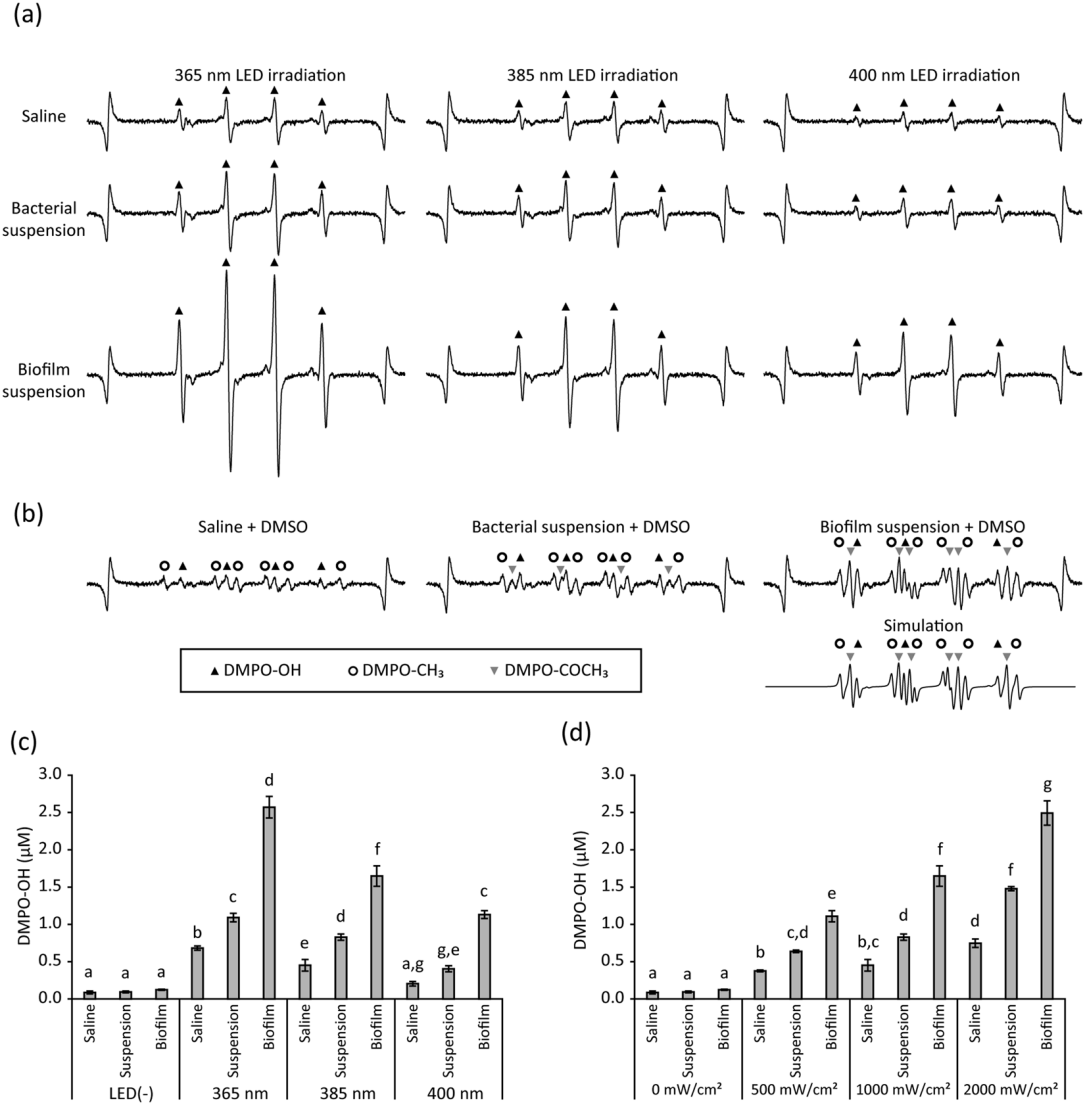
Analysis of hydroxyl radicals generated by LED irradiation of bacterial and biofilm suspensions. Hydroxyl radicals were qualitatively and quantitatively analysed using electron spin resonance (ESR)-spin trapping technique using 5,5-dimethyl-1-pyrroline *N*-oxide (DMPO) as a spin trap agent. (**a**) Representative ESR spectra of DMPO-OH (spin adduct of hydroxyl radical: closed triangle) generated using each condition (irradiance: 1000 mW/cm^2^, viable bacterial counts: 10^7^ CFU/sample, treatment time: 1 min). The peaks at both ends represent Mn^2+^ used as an internal standard. (**b**) Representative ESR spectra recorded when DMSO was added to the reaction system (365 nm LED irradiation of sample at 1000 mW/cm^2^). DMPO-OH, DMPO-CH_3_ (spin adduct of methyl radical: open circle) and DMPO-COCH_3_ (spin adduct of acetyl radical: inverse triangle) were detected. Computer simulation was performed using hyper fine coupling constants described in the text. (**c**) Yield of DMPO-OH generated by the LED irradiation of samples at different wavelengths (irradiance: 1000 mW/cm^2^, treatment time: 1 min). (**d**) Yield of DMPO-OH generated by the LED irradiation of samples at different irradiances (wavelength: 385 nm, treatment time: 1 min). Values and error bars in (**c**) and (**d**) indicate the mean and standard deviation, respectively. Different letters above the columns in (**c**) and (**d**) refer to significant differences (*p* < 0.05) between different groups.

When saline, the bacterial suspension, and the biofilm suspension (without CA) were exposed to LED light, DMPO-OH was also detected (Fig. 5a). The addition of DMSO to the reaction mixture resulted in the generation of DMPO-CH_3_ and a decrease in the intensity of DMPO-OH (Fig. 5b). In addition, DMPO-COCH_3_ (spin adduct of acetyl radical, hfcc: a_N_ = 1.52 mT, a_H_ = 1.87 mT)^23^ was detected when the bacterial suspension and the biofilm suspension were irradiated in the presence of DMSO. The presence of DMPO-COCH_3_ was also confirmed by computer simulation using the hfcc listed above. Quantitative analysis revealed that the yield of DMPO-OH depended on the wavelength and irradiance of the LED light (Fig. 5c,d). In general, shorter wavelengths and higher irradiances resulted in larger yields. In addition, LED irradiation of the biofilm suspension resulted in larger yield of DMPO-OH than irradiation of saline and the bacterial suspension.

### Bactericidal effect of acetyl radicals

**Figure 6:**
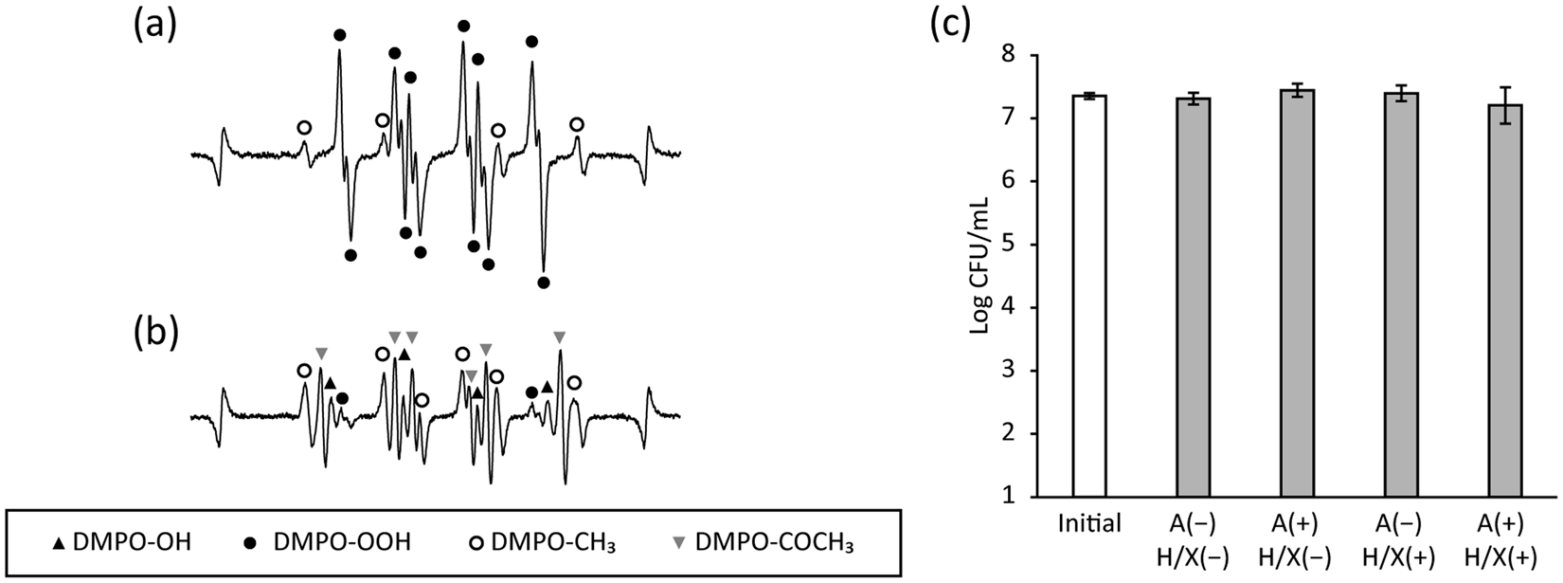
Generation of acetyl radicals by oxidation of acetaldehyde and evaluation of their antimicrobial activity. Superoxide anion radicals and acetyl radicals were qualitatively analysed using electron spin resonance (ESR)-spin trapping technique using 5,5-dimethyl-1-pyrroline N-oxide (DMPO) as a spin trap agent. The number of bacteria was determined after each treatment by standard plate counting. (**a**) Representative ESR spectra of DMPO-OOH (spin adduct of superoxide anion radicals: closed circle) generated by hypoxanthine (50 µM)/xanthine oxidase (50 mU/mL) reaction system. The peaks at both ends represent Mn^2+^ used as an internal standard. (**b**) Representative ESR spectra of DMPO-COCH_3_ (spin adduction of acetyl radicals: inverse triangle) generated as a result of oxidation of 50 mM acetaldehyde by superoxide anion radicals. (**c**) Antimicrobial activity of acetyl radicals. Values and error bars in (**c**) indicate the mean and standard deviation, respectively. A: 50 mM acetaldehyde, H/X: 50 µM hypoxanthine and 50 mU/mL xanthine oxidase.

Oxidation of 50 mM acetaldehyde by superoxide anion radicals generated by a hypoxanthine/xanthine oxidase (HPX/XOD) reaction system resulted in the generation of acetyl radicals (Fig. 6a,b). The HPX/XOD reaction system generated 7.4 µM superoxide anion radicals under the conditions used in the present study. When acetaldehyde was oxidised by the superoxide anion radicals, the peak intensity of the acetyl radicals was slightly higher than that of radicals generated by photo-irradiation of the biofilm suspension.

When planktonic *S*. *mutans* was added to the reaction system and treated for 4 min, none of the treatments tested (acetaldehyde alone, superoxide anion radicals, and acetyl radicals) resulted in a significant change in viable bacterial counts (Fig. 6c).

### Evaluation of transmittance and temperature change

**Figure 7:**
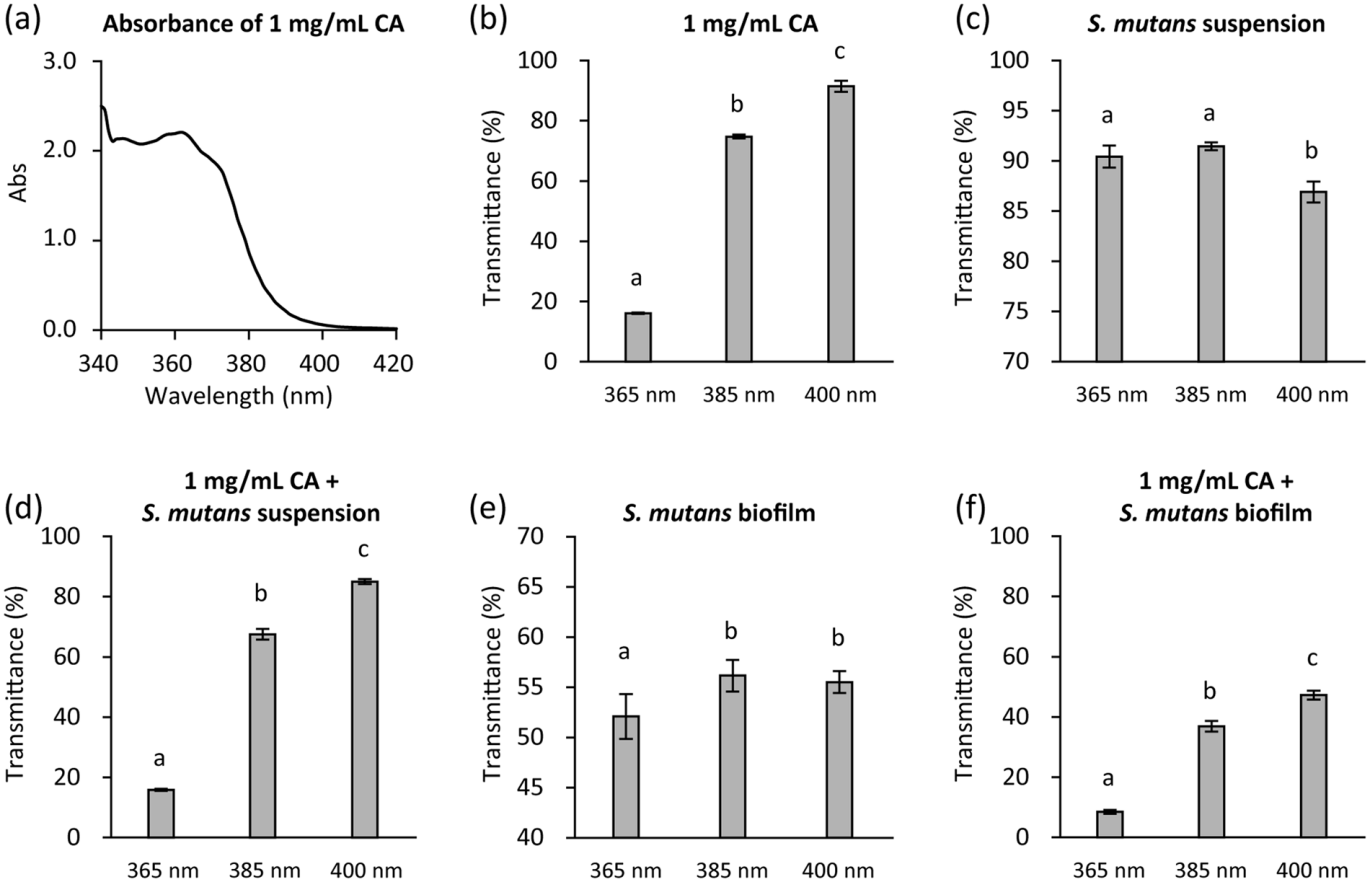
UV-Visible absorption spectrum of caffeic acid (CA) solution (**a**) and transmittance of LED light through each sample (**b**–**f**). The UV-visible absorption spectrum of 1 mg/mL CA prepared in pure water was obtained using a spectrophotometer. The transmittance was calculated as follows: transmittance, % = (power of transmitted light through the sample/blank) × 100. The test samples were 1 mg/mL CA, *S*. *mutans* suspension with 5 × 10^7^ CFU/mL, *S*. *mutans* biofilms, and combinations thereof. Values and error bars indicate the mean and standard deviation, respectively. Different letters above the columns refer to significant differences (*p* < 0.05) between different groups.

The UV-visible absorption spectrum of 1 mg/mL CA indicated a peak at 362 nm (Fig. 7a). Accordingly, the transmittance of light through the 1 mg/mL CA solution was in the order of 365 nm < 385 nm < 400 nm (Fig. 7b). The bacterial suspension also slightly inhibited the transmittance of light (Fig. 7c). Consequently, transmittance through the mixture of 1 mg/mL CA and the bacterial suspension was 16% for 365 nm, 68% for 385 nm, and 85% for 400 nm (Fig. 7d). Biofilms formed on the bottom of the wells also reduced light transmittance by almost 50% (Fig. 7e). The transmittance of light through the biofilm was significantly lower at 365 nm than at 385 and 400 nm. As a result, at 365, 385, and 400 nm, values for light transmittance through the well containing 1 mg/mL CA and the biofilm were 9, 37, and 47%, respectively (Fig. 7f).

During LED irradiation, the temperature of the CA solution, which was initially adjusted to 25 °C, increased with time (data not shown). When different wavelengths were compared using the same irradiance of 1000 mW/cm^2^, LED irradiation at 365 nm resulted in the highest temperature (38 °C) after 4 min. Similarly, when the effect of irradiance was compared using LED light with the same wavelength of 385 nm, LED irradiation at 2000 mW/cm^2^ resulted in the highest temperature (42 °C).

## Discussion

The present study demonstrated that the bactericidal activity of photo-irradiated CA was enhanced by the application of UV and by increasing the irradiance and treatment time as hypothesized, whereas the effect of CA concentration was limited. Thus, the hypothesis was partly rejected. LED irradiation of a 1-mg/mL CA solution at wavelength of 385 nm and an irradiance of 2000 mW/cm^2^ resulted in the best bactericidal effect against biofilm-forming *S*. *mutans*. The bacteria were killed with a >5-log reduction in viability within 4 min of treatment. Therefore, this antimicrobial technique, based on photo-irradiation of a CA solution, is suggested to be applicable as a novel adjunct chemotherapy for preventing and treating dental caries.

The experimental *S*. *mutans* biofilm model used herein was established in our previous study^11^. The hydroxyapatite disks, a substitute for tooth enamel, were first treated with artificial saliva to create a pellicle on the surface. Subsequently, the disks were incubated with an *S*. *mutans* suspension using gentle rotation to simulate oral shear stress. It was demonstrated that the biofilm formed using such conditions was composed of multiple bacterial cell layers with EPS and exhibited acquired resistance to antibiotics^11^. In the present study, the same experimental biofilm model was prepared and tested.

The wavelength of LED light significantly influenced bactericidal activity against *S*. *mutans* biofilms for both CA(−)L(+) and CA(+)L(+) treatments. For CA(−)L(+), a shorter wavelength resulted in a greater bactericidal effect. This finding is in accordance with a previous study that demonstrated the wavelength-dependent bactericidal activity of UV light^11, 16^. As expressed by the equation *E* = *hc/λ*, where *E* is energy, *h* is Planck’s constant, *c* is the speed of light, and *λ* is wavelength, photon energy is inversely proportional to wavelength. Thus, photon energy for light with a wavelength of 365 nm is highest, and would enhance the water photolysis reaction. ESR analysis demonstrated that of the wavelengths tested, 365 nm LED irradiation generated larger yields of hydroxyl radicals than any other wavelength when using the CA(−)L(+) treatment condition. As such, this resulted in the highest bactericidal effect against *S*. *mutans* biofilms.

Using the CA(+)L(+) treatment condition, 385 nm LED irradiation exerted the highest bactericidal effect against *S*. *mutans* biofilms. According to the absorption spectrum, the peak absorbance of CA solution was at 362 nm. This resulted in the lowest transmittance of the 365 nm LED light through the CA solution. Furthermore, the transmittance of this light through the biofilm was significantly lower than that with 385 and 400 nm LED light. Consequently, transmittance through a combination of the CA solution and the biofilm was 9%, whereas that with 385 and 400 nm LED light were 37% and 47%, respectively. Although ESR analysis showed that CA(+)L(+) treatment at an LED wavelength of 365 nm generated the largest yield of hydroxyl radicals, this reflected the total yield in the solution but not the effective dose for treating the biofilm. Considering that irradiance decreases with the depth of the solution as a result of absorption, hydroxyl radical generation would likely be higher near the surface of the CA solution than at the bottom where the biofilm sample was immersed. Thus, the yield of hydroxyl radicals generated by CA(+)L(+) at 365 nm might be lower near the biofilm, when compared to that generated by CA(+)L(+) at 385 nm, which would result in lower bactericidal activity for the former treatment compared to the latter.

Regarding the influence of irradiance, higher levels yielded greater bactericidal effects for both conditions, namely CA(−)L(+) and CA(+)L(+). This could be due to increased yield of hydroxyl radicals induced by augmentation of irradiance as shown by ESR analysis. The increase in irradiance would enhance both photo-oxidation reactions of CA and water photolysis. Comparing the bactericidal activities of CA(−)L(+) and CA(+)L(+) performed at the same irradiance, the latter showed higher bactericidal effect. This might also be explained by the larger yield of hydroxyl radicals generated by CA(+)L(+), compared to that with CA(−)L(+).

The influence of the CA concentration, in the range of 0.25–2 mg/mL, on bactericidal activity of CA(+)L(+) was limited. As previously reported^14^, the bactericidal effect of photo-irradiated polyphenols can be determined by the relationship between anti-oxidative and pro-oxidative activity. Polyphenols including CA are noteworthy for their anti-oxidative activity^24, 25^. Thus, excessive amounts of CA might scavenge the hydroxyl radicals generated by photo-irradiation. Another possible explanation of this observation is that the concentration of CA might be excessive compared to the amount of CA that is photo-oxidized in the reaction system. In fact, 1 mg/mL CA corresponds to 5.6 mM, which was much larger than the yield of hydroxyl radicals (<3 µM/min) generated by CA(+)L(+). This suggests that photo-irradiation is the rate limiting factor, and as such, the concentration of CA tested in this study had little effect on the bactericidal activity.

Viable bacterial counts in biofilms were reduced by CA(+)L(+) treatment in a time dependent manner. During the first minute, the largest reduction (3-log reduction) was observed. Although the reduction rate subsequently diminished, a 4-min treatment resulted in a >5-log reduction in viability, and demonstrating a higher bactericidal effect at each time point, compared to that with CA(−)L(+) treatment. Considering the rise in temperature with irradiation time, the additional thermal energy would somewhat accelerate hydroxyl radical generation as previously reported^26^. Nonetheless, the reduction rate in viable counts decreased with time. This might be due to the attenuation of the bactericidal activity in relation to the location of bacteria within the biofilm. It is assumed that the deeper the bacteria are in the biofilm, the more they will be protected by the barrier effect of EPS, which would prevent both light and CA penetration. Thus, CA(+)L(+) would exert a higher bactericidal effect near the surface of the biofilm than in the deeper areas, resulting in the observed decrease in reduction rate over time, even though the yield of hydroxyl radicals increased with rising temperatures.

Planktonic *S*. *mutans* was also susceptible to CA(+)L(+) as previously reported^13^. The bactericidal effect depended on the wavelength, similar to that observed for biofilm bactericidal assays (i.e. viable counts decreased in the following order: 385 nm > 365 nm > 400 nm). As in the case of the biofilm, since 365 nm LED light would be absorbed by the CA solution, 385 nm LED light resulted in a higher bactericidal effect. However, as planktonic bacteria existed homogeneously in suspension, the influence of light absorption on bactericidal effect might be smaller than that for the biofilm. Indeed, CA(+)L(+) at 365 and 385 nm was shown to reduce viable counts below the detection limit after a 4-min treatment. When planktonic bacteria and biofilm-forming bacteria were treated with CA(+)L(+) under the same conditions, the former showed higher susceptibility to treatment than the latter. Since hydroxyl radicals do not diffuse over long distance^27^, CA must be present inside or in the vicinity of the bacterial cells to exert its antimicrobial activity via hydroxyl radical formation upon photo-irradiation. Thus, in the case of the biofilm, the EPS might prevent CA penetration resulting in higher resistance for biofilm-forming bacteria. However, considering the reduction in bacterial viable counts after CA(+)L(+) treatment, EPS did not completely inhibit penetration, and CA likely reached the bacterial cells.

In contrast, planktonic bacteria exhibited a higher resistance to CA(−)L(+) than biofilm-forming bacteria. This finding is in accordance with a previous study^11^. It was discussed that bacterial cells in the biofilm might be more vulnerable than those in suspension that are freshly prepared from culture. However, in the present study, ESR analysis showed that LED irradiation of the biofilm suspension generated hydroxyl radicals even though CA was not present in the reaction mixture. When DMSO was added to the reaction system, a signal for DMPO-CH_3_ emerged, whereas the peak intensity for DMPO-OH decreased. It is known that DMPO-CH_3_ is produced through the oxidation of DMSO by hydroxyl radicals^28^, indicating that DMPO-OH detected by ESR analysis represents hydroxyl radical generation^15, 16^ but not nucleophilic addition of water^29^. Thus, the biofilm might contain substances that would contribute to the generation of hydroxyl radicals upon photo-irradiation. In addition, the hydroxyl radicals generated by photo-irradiation of the biofilm might contribute to lethal oxidative damage to the bacterial cells. Although the yield was lower than that for the biofilm suspension, photo-irradiation of the bacterial suspension also generated hydroxyl radicals. These findings indicate that such substances might be contained in both bacterial cells and the extracellular matrix. Identification of the substance that contributes to these antimicrobial effects (against the biofilm) upon photo-irradiation could lead to the development of novel inactivation techniques for biofilms.

ESR analysis demonstrated that the photo-irradiation of bacterial and biofilm suspensions generated acetyl radicals in the presence of DMSO. Nakao *et al*. showed that acetyl radicals were generated through the oxidation of acetaldehyde^23^. It has been reported that *Streptococcus* species including *S*. *mutans* produce acetaldehyde, though at a low yield^30^. In the absence of DMSO, acetyl radicals might not be trapped by DMPO because of the high rate constant for the reaction between hydroxyl radicals and DMPO (k = 3.4 × 10^9^ M^−1^s^−1^)^31^. Additional ESR analysis confirmed that oxidation of acetaldehyde by superoxide anion radicals resulted in the generation of acetyl radicals. Thus, the bactericidal effect of acetyl radicals was tested using this reaction system. The results showed that viable counts of planktonic *S*. *mutans* were not affected by the treatment with acetyl radicals. Hence, it is suggested that acetyl radicals generated by photo-irradiation of the biofilm are not involved in the bactericidal activity against *S*. *mutans* biofilms.

Within the limitations of the present study, it is suggested that LED irradiation of 1 mg/mL CA at a wavelength of 385 nm and an irradiance of 2000 mW/cm^2^ for 4 min exerts the most prominent bactericidal effect against *S*. *mutans* biofilms. Since even a 1-min treatment resulted in a 3-log reduction in viable bacterial counts for the biofilm, treatment times of upwards of 4 min might not be necessary to achieve significant effects. Furthermore, even if the bacteria survive the antimicrobial treatment, their regrowth will be suppressed by the damage caused by hydroxyl radical treatment as previously reported^32^. Thus, treatment time should be further evaluated and optimized from a clinical perspective. Regarding this technique, the bactericidal effect on biofilms was additionally confirmed by CLSM. However, it should be noted that the biofilm was not removed from the surface of the specimen through photo-irradiation treatment. To effectively reduce virulence, the antimicrobial treatment should be performed with mechanical removal of the biofilm. Mechanical means will considerably reduce the amount of bacteria and will destroy the structure of biofilm. It is reasonable to assume that the antimicrobial chemotherapy may become more efficient against mechanically treated biofilm than that against intact biofilm. Overall, this technique is relatively simple consisting of light and an edible polyphenol, and the temperature increase of up to 42 °C during photo-irradiation is acceptable. Therefore, photo-irradiation of CA has the potential to be applied as an inexpensive antimicrobial therapy, in combination with mechanical removal of pathogens, to prevent and treat dental caries.

## Materials and Methods

### Reagents

Reagents were purchased as follows: CA was from Tokyo Chemical Industries (Tokyo, Japan). 4-hydroxy-2,2,6,6,-tetramethylpiperidine *N*-oxyl (TEMPOL) and HPX were from Sigma-Aldrich (St. Louis, MO, USA). DMPO and XOD were from Labotec (Tokyo, Japan). Bovine serum albumin, α-amylase, lysozyme, mucin, catalase, DMSO, and acetaldehyde were from Wako Pure Chemicals Industries (Osaka, Japan). All other reagents used were analytical grade.

### Experimental biofilm model of *S*. *mutans*

Biofilm formation was induced according to a previous study^11^. In brief, the biofilm was formed on hydroxyapatite disks of a diameter and thickness of 5 and 2 mm, respectively (CellYard HA pellet D5-T2, HOYA, Tokyo, Japan). Before each experiment, the disks were polished with silicon carbide papers up to #1500, and were autoclaved at 121 °C for 15 min.

The test strain was *S*. *mutans* JCM 5705, provided from the Japan Collection of Microorganisms (RIKEN BioResource Center, Wako, Japan). Cultures were anaerobically grown using AneroPack (Mitsubishi Gas Chemical Company, Tokyo, Japan) in brain heart infusion (BHI) broth (Becton Dickinson Labware, Franklin Lakes, NJ) at 37 °C for 24 h. From this culture, a bacterial suspension was prepared in sterile saline. The concentration was adjusted to approximately 10^8^ CFU/mL.

Autoclaved disks were incubated with 500 µL of artificial saliva in a 48-well plate at 37 °C for 1 h. The artificial saliva was prepared according to previous studies^33, 34^. Specifically, phosphate buffered saline was supplemented with 40 µg/mL of bovine serum albumin, 1 mg/mL of α-amylase, 10 µg/mL of lysozyme, and 850 mg/mL of mucin. After incubation, artificial saliva was removed, and the wells were filled with 1000 µL of BHI broth supplemented with 1% sucrose and 100 µL of the bacterial suspension (inoculum size = 10^7^ CFU). Subsequently, the disks were incubated anaerobically at 37 °C for 24 h under gentle rotation at 200 rpm (MicroMixer E-36, TAITEC, Koshigaya, Japan). Before each assay, the biofilm specimens were gently washed twice with saline to remove unattached bacteria.

### Bactericidal effect of photo-irradiated CA on *S*. *mutans* biofilm

CA solution was prepared at time of use by dissolving the CA powder in pure water to be 2 mg/mL (corresponding to 11.1 mM). The tube containing CA was placed in hot water (90 °C) and vortexed to completely dissolve the CA. It was previously confirmed that CA does not decompose with heating^13^. When applicable, CA solution was diluted with pure water to 0.25, 0.5, and 1 mg/mL.

Biofilms were treated with either a single or combined treatment of LED irradiation and immersion in CA solution. As stated previously, the treatment groups were CA(−)L(−), CA(+)L(−), CA(−)L(+), or CA(+)L(+). CA(+) specimens were immersed in 500 µL of the CA solution in one well of a 48-well plate, whereas CA(−) specimens were immersed in 500 µL of pure water. In addition, the L(+) specimens were exposed to light-irradiation, whereas the L(−) specimens were kept in a light-shielding box. The light source was an LED spot-curing device (OmniCure LX400; Lumen Dynamics Group, Mississauga, ON, Canada).

First, the influence of light wavelength on bactericidal activity was evaluated. The L(+) specimens were irradiated with 365, 385, or 400 nm LED light at an irradiance of 1000 mW/cm^2^ for 4 min. The CA solution was used at a concentration of 1 mg/mL. Second, the influence of irradiance was evaluated. The L(+) specimens were irradiated with 385 nm LED light at irradiances of 500, 1000, or 2000 mW/cm^2^ for 4 min. The irradiances tested in the present study were comparable to or less than those of LED curing light used in oral cavity^35^. Thus, it is assumed that the irradiances could be safely applied in caries treatment. The CA solution was used at a concentration of 1 mg/mL. Third, the influence of CA concentration was evaluated. The specimens were immersed in CA solution prepared at concentrations of 0, 0.25, 0.5, 1, or 2 mg/mL and irradiated with 385 nm LED light at an irradiance of 2000 mW/cm^2^ for 1 min. Lastly, the influence of treatment time was evaluated. The specimens were immersed in pure water [CA(−)L(+)] or 1 mg/mL CA solution [CA(+)L(+)], and were irradiated with 385 nm LED light at an irradiance of 2000 mW/cm^2^ for 0, 1, 2, and 4 min.

After each treatment, the specimens were gently washed twice with saline. The biofilm was swabbed using a sterile cotton swab. The collected bacteria were suspended in 200 µL of saline, and the mixture was serially diluted 10-fold with saline, and 10 µL of the dilution was plated on BHI agar (Becton Dickinson Labware). The agar plates were cultured anaerobically at 37 °C for 48 h, which was followed by colony enumeration. All tests were performed using six independent assays.

The biofilms treated with CA(−)L(−), CA(+)L(−), CA(−)L(+), and CA(+)L(+) were subjected to CLSM. CA was used at 1 mg/mL and 385 nm LED irradiation was performed at an irradiance of 2000 mW/cm^2^ for 4 min. After treatment followed by washing, the biofilm was fluorescently stained with a FilmTracer LIVE/DEAD Biofilm Viability Kit (Thermo Fisher Scientific, Waltham, MA) containing SYTO9 and PI for 20 min at room temperature, according to the manufacturer’s instructions. Dead bacteria were stained by only PI, whereas both dead and living bacteria were stained by SYTO9. The biofilm was imaged by CLSM (TCS-SPE; Leica Microsystems, Wetzlar, Germany).

### Bactericidal effect of photo-irradiated CA on planktonic *S*. *mutans*

A bacterial suspension of *S*. *mutans* was prepared in saline from a culture grown anaerobically in BHI broth for 24 h as described above. In 96-well plates, 100 µL of bacterial suspension was mixed with the same volume of 2 mg/mL CA. Thus, the final concentration of CA was 1 mg/mL and viable bacterial count was 10^7^ CFU/well (concentration: 5 × 10^7^ CFU/mL). The suspension was treated with CA(−)L(−), CA(+)L(−), CA(−)L(+), or CA(+)L(+). L(+) samples were irradiated with 365, 385, or 400 nm LED light at an irradiance of 1000 mW/cm^2^. CA(+)L(+) treatment was performed for 1, 2, and 4 min, whereas other treatments were performed only for 4 min. After treatment, 180 µL of sample was mixed with 20 µL of catalase solution (25,000 U/mL) to terminate the effect of H_2_O_2_ generated by photo-irradiation of CA^13^. Viable bacterial counts were determined as described above. All tests were performed using six independent assays.

### Analysis of hydroxyl radical generation

The yield of hydroxyl radicals generated by photo-irradiation of CA solution was analysed using an ESR-spin trapping technique according to a previous study^13^. Briefly, 100 µL of CA solution was mixed with 50 µL of DMPO and 50 µL of pure water to reach final concentrations of 1 mg/mL for CA and 300 mM for DMPO. Subsequently, the sample was irradiated with 365, 385, or 400 nm LED light at an irradiance of 1000 mW/cm^2^ for 60 s, or with 385 nm LED light at an irradiance of 500, 1000, or 2000 mW/cm^2^ for 60 s. To investigate whether DMPO-OH was derived from hydroxyl radical generation, an additional assay was performed in which 50 µL of 14 M DMSO (final concentration: 3.5 M), an authentic hydroxyl radical scavenger, was added to the reaction system instead of pure water, and the mixture was irradiated with 385 nm LED light at 1000 mW/cm^2^ for 60 s. The ESR spectrum of the mixture was recorded using an X-band ESR spectrometer (JES-FA-100, JEOL, Tokyo, Japan). The measurement conditions for ESR were as follows: field sweep, 332.07–342.07 mT; field modulation frequency, 100 kHz; field modulation width, 0.1 mT; amplitude, 200; sweep time, 2 min; time constant, 0.03 s; microwave frequency, 9.420 GHz; microwave power, 4 mW. TEMPOL (5 µM) was used as a standard to calculate the concentration of DMPO-OH. Qualitative and quantitative analyses were performed using software (Digital Data Processing, JEOL). All tests were performed using three independent assays.

Similarly, saline, bacterial suspensions, and biofilm suspensions, with or without LED irradiation, were subjected to ESR-spin trapping analysis. Bacterial suspensions of *S*. *mutans* were prepared in saline as described above. Regarding biofilm suspensions, hydroxyapatite disks on which biofilms were formed were immersed in 200 µL of saline, and biofilms were detached by scraping and suspended using a disposable plastic stick. The resultant sample (100 µL) was mixed with 50 µL of DMPO and 50 µL of pure water. Thus, the final concentration of DMPO was 300 mM and the viable count was 10^7^ CFU. The mixture was irradiated with a 365, 385, or 400 nm LED light at an irradiance of 1000 mW/cm^2^ for 60 s, or with 385 nm LED light at an irradiance of 500, 1000, or 2000 mW/cm^2^ for 60 s. An additional assay was performed, in which 50 µL of 14 M DMSO (final concentration: 3.5 M) was added to the reaction system instead of pure water, and the mixture was irradiated using 365 nm LED light at 1000 mW/cm^2^ for 60 s. ESR analysis was performed as described above. All tests were performed using three independent assays.

### Bactericidal effect of acetyl radicals

Acetaldehyde, HPX, and XOD were diluted or dissolved in pure water to 200 mM, 200 µM, and 400 mU/mL, respectively. The reagents were mixed in the following order: 50 µL of acetaldehyde, 50 µL of HPX, 25 µL of 14 M DMSO, 50 µL of 1200 mM DMPO, and 25 µL of XOD resulting in final concentrations of 50 mM, 50 µM, 1.75 M, 300 mM, and 50 mU/mL, respectively. After addition of XOD, the mixture was left for 1 min, and then subjected to ESR analysis as described above. ESR analysis for the reaction mixture without acetaldehyde was also conducted. All tests were performed using three independent assays.

A bacterial suspension of *S*. *mutans* prepared at 8 × 10^7^ CFU/mL was added to the reaction system (final concentration: 2 × 10^7^ CFU/mL) instead of DMPO. The bacteria were treated in the reaction mixture for 4 min. Viable bacterial counts were determined as described above. All tests were performed using six independent assays.

### Evaluation of light transmittance and temperature change

The UV-visible absorption spectrum of 1 mg/mL CA was obtained using a spectrophotometer (Gene Quant 1300; GE Healthcare, Bukinghamshire, UK). Transmittance through the samples for 365, 385, and 400 nm LED light at an irradiance of 1000 mW/cm^2^ was analysed by measuring the power of the light. The power of transmitted light through an empty well of a 48-well plate was measured using a power meter (FieldMate, Coherent, Santa Clare, CA) and this was regarded as the blank. Then, 300 µL of CA solution, *S*. *mutans* suspension, or a mixture of CA solution and the bacterial suspension was added to the wells to achieve final concentrations of 1 mg/mL CA and 5 × 10^7^ CFU/mL of bacteria. Subsequently, the power of transmitted light through the well containing the sample was measured. In addition, *S*. *mutans* biofilms were prepared at the bottom of wells of a 48-well plate in the same manner that was used for biofilm formation on hydroxyapatite specimens. The power of transmitted light through the biofilm with or without 300 µL of 1 mg/mL CA was measured. The transmittance was calculated as follows: transmittance, % = (power of transmitted light through the sample/blank) × 100.

The temperature change during LED irradiation of the CA solution was monitored using a platinum resistance thermometer (TUSB-S01PT2Z, Turtle Industry, Tsuchiura, Japan). The thermo-sensor was placed in a well of a 48-well plate containing 1 mg/mL CA. The solution with an initial temperature of 25 °C was irradiated with 365, 385, or 400 nm LED light at an irradiance of 1000 mW/cm^2^ for up to 4 min, or with 385 nm LED light at an irradiance of 500, 1000, or 2000 mW/cm^2^ for up to 4 min.

### Statistical analyses

Statistical significances (p < 0.05) for bactericidal assays (CFU values), hydroxyl radical yields, and transmittance values were assessed by either a Student’s *t*-test for pairwise comparisons or a one-way analysis of variance followed by the Tukey-Kramer honestly significant difference test for multiple comparisons using JMP Pro 11.0 software (SAS Institute, Cary, NC). Analyses for CFU values were performed on logarithmically converted viable counts. In the absence of detectable colonies, the half value of the detection limit (10 CFU) was assumed for statistical analysis.

### Data availability

The datasets generated and/or analysed during the current study are available from the corresponding author on reasonable request.
